# Myocardial bridging in obstructive hypertrophic cardiomyopathy: a risk factor for myocardial fibrosis

**DOI:** 10.1186/s12916-024-03301-6

**Published:** 2024-02-27

**Authors:** Changpeng Song, Shengwei Wang, Xinli Guo, Manyun Huang, Xinxin Zheng, Jie Lu, Keshan Ji, Shihua Zhao, Jingang Cui, Shuiyun Wang, Xiaohong Huang

**Affiliations:** 1 Department of Special Medical Treatment Center in Fuwai Hospital, National Center for Cardiovascular Diseases, Chinese Academy of Medical Sciences and Peking Union Medical College, 167 Beilishi Road, Beijing, 100037 People’s Republic of China; 2 Department of Cardiovascular Surgery Center, Beijing Anzhen Hospital, Capital Medical University, Beijing Institute of Heart, Lung and Blood Vascular Diseases, Beijing, People’s Republic of China; 3 Department of Magnetic Resonance Imaging in Fuwai Hospital, National Center for Cardiovascular Diseases, Chinese Academy of Medical Sciences and Peking Union Medical College, Beijing, People’s Republic of China; 4 Department of Cardiovascular Surgery in Fuwai Hospital, National Center for Cardiovascular Diseases, Chinese Academy of Medical Sciences and Peking Union Medical College, Beijing, People’s Republic of China

**Keywords:** Hypertrophic cardiomyopathy, Myocardial bridging, Fibrosis, Survival

## Abstract

**Background:**

Myocardial bridging (MB) is common in patients with hypertrophic cardiomyopathy (HCM). There are sparse data on the impact of MB on myocardial fibrosis in HCM. This study was designed to evaluate the relationship between MB and myocardial fibrosis in patients with obstructive HCM.

**Methods:**

In this cohort study, retrospective data were collected from a high-volume HCM center. Patients with obstructive HCM who underwent septal myectomy and preoperative cardiac magnetic resonance (CMR) were screened from 2011 to 2018.

**Results:**

Finally, 492 patients were included in this study, with an average age of 45.7 years. Of these patients, 76 patients had MB. MB occurred mostly in the left anterior descending artery (73/76). The global extent of late gadolinium enhancement (LGE) was correlated with the degree of systolic compression (*r* = 0.33, *p* = 0.003). Multivariable linear regression analysis revealed that the degree of systolic compression was an independent risk factor for LGE (*β* = 0.292, *p* = 0.007). The LGE fraction of basal and mid anteroseptal segments in patients with severe MB (compression ratio ≥ 80%) was significantly greater than that in patients with mild to moderate MB (compression ratio < 80%). During a median follow-up of 28 (IQR: 15–52) months, 15 patients died. Kaplan–Meier analysis did not identify differences in all-cause death (log-rank *p* = 0.63) or cardiovascular death (log-rank *p* = 0.72) between patients undergoing MB-related surgery and those without MB.

**Conclusions:**

MB with severe systolic compression was significantly associated with a high extent of fibrosis in patients with obstructive HCM. Concomitant myotomy or coronary artery bypass grafting might provide excellent survival similar to that of patients without MB. Identification of patients with severe MB and providing comprehensive management might help improve the prognosis of patients with HCM.

## Background

Myocardial fibrosis is one of the major histologic hallmarks of hypertrophic cardiomyopathy (HCM), and extensive fibrosis is an independent risk factor for poor prognosis [1–3]. Myocardial fibrosis in HCM could be partly attributable to myocardial ischemia resulting from a mismatch between myocardial oxygen supply and demand. Left ventricular output tract obstruction (LVOTO) with high left ventricular wall stress could worsen ischemia [1–3].

Myocardial bridging (MB), a congenital anomaly, is characterized as a segment of the epicardial coronary artery that traverses through the myocardium, resulting in systolic compression of the tunneled artery [4]. MB is common in patients with hypertrophic cardiomyopathy, with a prevalence of 11–37% [5, 6]. Previous studies have shown that MB does not affect the long-term prognosis of HCM [7]. However, some studies reported that MB significantly altered diastolic flow and reduced coronary flow in patients with HCM, which could be responsible for myocardial ischemia and further affect myocardial fibrosis [8, 9].

Contrast-enhanced cardiovascular magnetic resonance (CMR) imaging with late gadolinium enhancement (LGE) is a noninvasive and reliable imaging study that uses multiplanar imaging to comprehensively assess global and focal myocardial fibrosis [10]. To date, there are sparse data on the impact of MB on myocardial fibrosis in HCM. The main purpose of this study was to evaluate the relationship between MB and myocardial fibrosis in patients with obstructive HCM.

## Methods

### Population selection

**Fig. 1:**
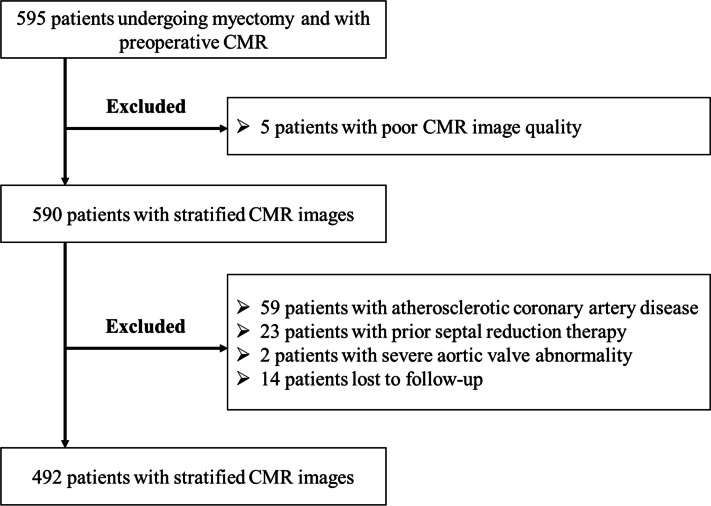
Flowchart of patient inclusion. Finally, 492 patients were enrolled in this analysis. CMR, cardiac magnetic resonance

To minimize selection bias, only obstructive HCM patients were included in this retrospective study (Fig. 1). The majority of patients undergoing myectomy underwent coronary angiography/computerized tomography angiography and CMR, while only a small proportion of patients without LVOTO underwent these imaging tests. Five hundred and ninety-five patients with obstructive HCM who underwent septal myectomy and preoperative CMR were screened between 2011 and 2018 at Fuwai Hospital. The diagnosis of HCM was based on the presence of myocardial hypertrophy (maximum wall thickness ≥ 15 mm or 13 mm in patients with a family history of HCM) in the absence of any other cause of cardiac hypertrophy [8]. Septal myectomy was performed in patients with HCM with drug-refractory symptoms and a maximum LVOT gradient or mid-ventricular gradient ≥ 50 mmHg at rest or with physiologic provocation. The exclusion criteria were (a) CMR images that were not clear enough to analyze, (b) no coronary angiography, (c) a history of coronary artery disease or septal reduction therapy, and (d) a history of other cardiac surgery. The study was approved by the Ethics Committees of Fuwai Hospital, Chinese Academy of Medical Sciences, and performed in accordance with the Declaration of Helsinki. All patients provided written informed consent.

### MB analysis

All patients in this study underwent preoperative coronary angiography. In this study, MB was defined as maximal systolic compression of the tunneled artery of ≥ 50%. Once an MB was detected, the location, length, and maximal degree of compression were described. The depth of the overlying myocardium was recorded in those patients who underwent myocardial myotomy at the same time as myectomy.

### CMR protocols and image analysis

CMR imaging was performed with a 1.5 Tesla cardiac magnetic scanner (Magnetom Avanto, Siemens Medical Solutions, Erlangen, Germany) or a 3.0-T scanner (Discovery MR750, GE Healthcare). Steady-state free-precession cine images were obtained in a four-chamber view and two-chamber view and continuous short-axis sections from the mitral annulus to the left ventricular (LV) apex. Typical imaging parameters included section thickness, 8 mm; section gap, 2 mm, for a 3.0 T scanner, repetition time 2.9–3.4 ms, echo time 1.5–1.7 ms, for a 1.5 T scanner repetition time 3.0 ms, echo time 1.1 ms. A phase-sensitive inversion-recovery turbo fast low-angle shot sequence was used for the LGE images. LGE images were obtained 10–15 min after the intravenous administration of 0.15 mmol/kg gadolinium contrast agent. All cardiac MRI images were analyzed using commercial imaging workstations (Siemens Medical Systems or Circle Cardiovascular Imaging). The left atrial dimension, LV end-diastolic diameter, LV end-diastolic volume (LVEDV), LV mass, and LV ejection fraction (LVEF) were measured by means of standard volumetric techniques [11]. LGE was assessed in the short-axis view and quantified using the full width half maximum method [12]. The extent of LGE was defined as the percentage of LV mass containing LGE relative to the total LV mass. Segmental results are shown as the American Heart Association 16-segment model [12].

### Histopathology

**Fig. 2:**
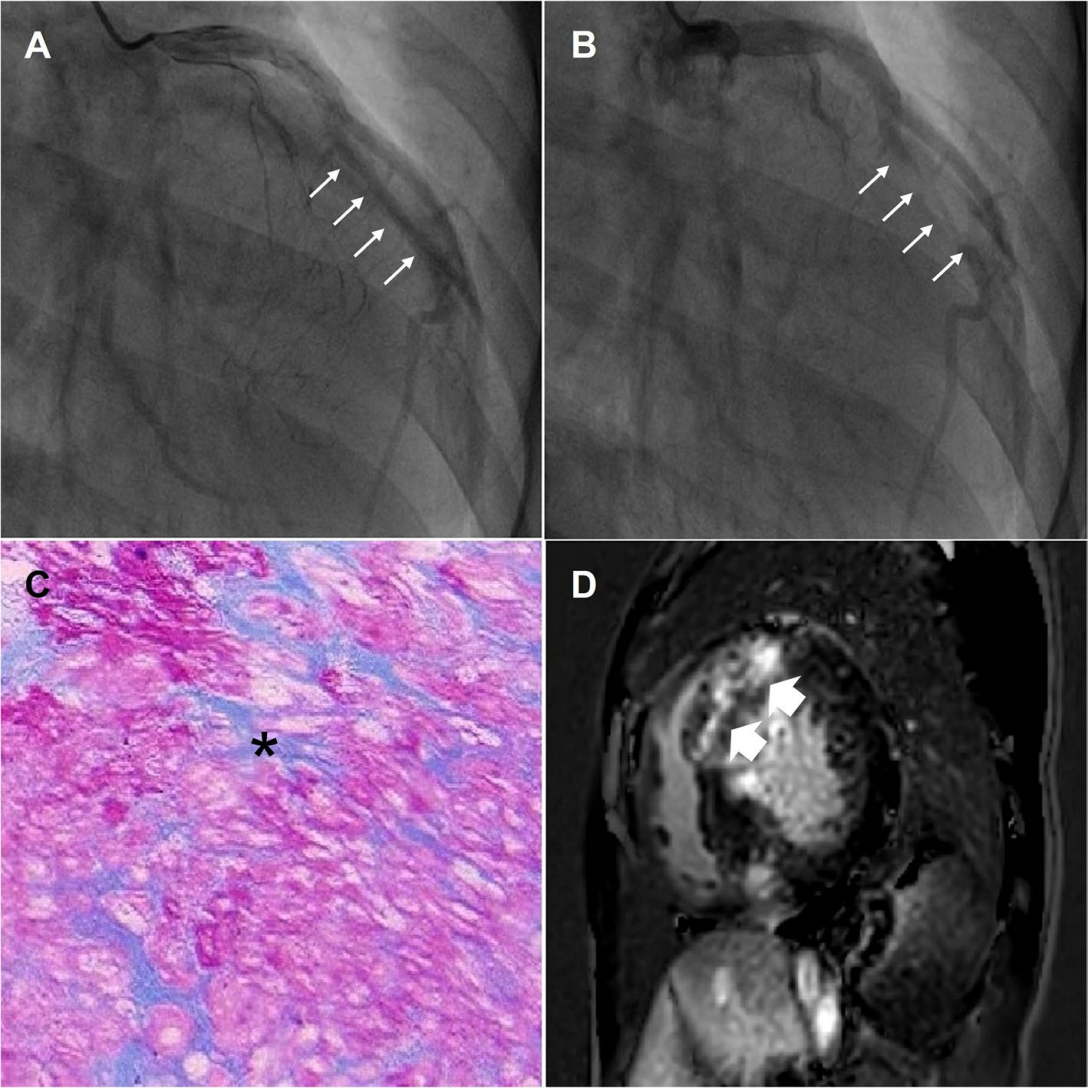
Myocardial fibrosis in a male patient with severe MB. Coronary angiography showed systolic compression of the left anterior descending coronary artery (LAD) (**A**, arrow) and almost complete recovery in diastole (**B**, arrow). Myocardial fibrosis is shown in the histological image (**C**, asterisk) and CMR images (**D**, arrow)

Myocardial specimens were obtained during septal myectomy in 137 patients. The myectomy tissue specimens were immediately fixed in 10% buffered formalin and subsequently embedded in paraffin. The samples were sectioned and stained with Masson’s trichrome stain to evaluate myocardial fibrosis. An automated image analysis protocol (using Image-Pro Plus 6.0) was used to determine the myocardial collagen content. The extent of myocardial fibrosis was expressed as the ratio of collagen-specific staining to the total area of the myocardium. The endocardium was excluded from the analysis (Fig. 2).

### Clinical outcomes and endpoints

Clinical data were collected. Follow-up data were obtained through telephone interviews, hospital records, and clinical visits from myectomy to June 2018. Patients who died were censored the same day. Survival analysis included all-cause and cardiovascular mortality.

### Statistical analysis

Continuous values are expressed as the means ± standard deviations or median (quartile). Continuous data were compared using Student’s *t* test or the Mann–Whitney *U* test. Categorical measures are presented as numbers (percentage) and were compared using the chi-square test or Fisher’s exact test. Linear regression was used to evaluate the relationship between the degree of systolic compression and the extent of LGE in patients with MB. Patients with MB were divided into 2 groups according to the median systolic compression (severe MB: systolic compression ratio ≥ 80% and mild to moderate MB: systolic compression ratio < 80%). The differences in the distribution of LGE among the three groups were compared (no MB, mild to moderate MB and severe MB). Kaplan–Meier survival analysis and the log-rank test were used for comparison of survival among the two groups (patients undergoing MB-related surgery and patients without MB). If values < 0.05, the differences were considered significant; all are reported as 2-sided. All analyses were performed using SPSS version 22.0 (IBM Corp, NY, USA) and GraphPad 7.10 (GraphPad Software, La Jolla, CA, USA).

## Results

### Study population

A total of 492 patients [288 males (58%)] were eventually enrolled in this study, with an average age of 45.7 years. Of these patients, 95 (19%) had hypertension, and 111 (23%) had syncope. The average LVOT gradient was 81.2 ± 27.3 mmHg. The median LGE percentage of the cohort was 8.1% (IQR: 3.6–16.1%) (Table 1).

**Table 1:** Demographic and clinical characteristics of study participants

Variables	All patients (*N* = 492)	Patients with MB (*N* = 76)	Patients without MB (*N* = 416)	*p* value
No. of males, *n*	288 (59%)	53 (70%)	235 (56%)	0.031
Age, years	45.7 ± 13.8	37.2 ± 13.6	47.2 ± 13.3	< 0.001
BMI, Kg/m^2^	24.9 ± 3.7	23.3 ± 3.8	25.3 ± 3.6	< 0.001
Dyspnea, *n*	394 (80%)	59 (78%)	335 (81%)	0.561
Chest pain, *n*	135 (27%)	21 (28%)	114 (27%)	0.967
Syncope, *n*	111 (23%)	22 (29%)	89 (21%)	0.147
NYHA III/IV, *n*	397 (81%)	59 (78%)	338 (81%)	0.462
Hypertension, *n*	95 (19%)	5 (7%)	90 (22%)	0.002
Diabetes mellitus, *n*	16 (3%)	1 (1%)	15 (4%)	0.301
Beta blocker, *n*	371 (75%)	59 (78%)	312 (75%)	0.624
CCB, *n*	53 (11%)	8 (11%)	45 (11%)	0.940
LAD, mm	42.1 ± 8.3	41.1 ± 8.4	42.3 ± 8.3	0.247
LVEDD, mm	45.7 ± 5.0	45.5 ± 4.9	45.8 ± 5.0	0.673
Maximal WT, mm	26.0 ± 7.7	26.7 ± 6.5	25.9 ± 7.9	0.377
LVEDVi, ml/m^2^	72.9 (62.5–87.0)	70.3 (62.1–87.6)	73.0 (62.6–87.0)	0.774
LV mass I, g/m^2^	90.5 (71.4–119.3)	92.2 (69.7–131)	90.1 (71.5–118.3)	0.359
LGE mass I, g/m^2^	7.3 (3.1–15.9)	8.7 (4.0–22.4)	7.0 (2.8–14.7)	0.022
LGE, % of LV mass	8.1 (3.6–16.1)	10.3 (5.1–19.1)	7.7 (3.3–15.5)	0.032
LVEF, %	71.3 ± 6.3	71.4 ± 5.7	71.3 ± 6.4	0.904
Maximal LVOTG, mmHg	81.2 ± 27.3	76.7 ± 30.0	82.0 ± 26.7	0.121

*BMI* Body mass index, *CCB* Calcium-channel blocker, *LAD* Left atrium dimension, *LGE mass I*, late gadolinium enhancement mass index, *LV* Left ventricular, *LVEDD* LV end-diastolic diameter, *LVEDVi* LV end-diastolic volume index, *LVEF* LV ejection fraction, *LV mass I*, left ventricular mass index, *LVOTG* LV outflow tract gradient, *NYHA* New York Heart Association

### Comparison of clinical variables between patients with and without MB

**Fig. 3:**
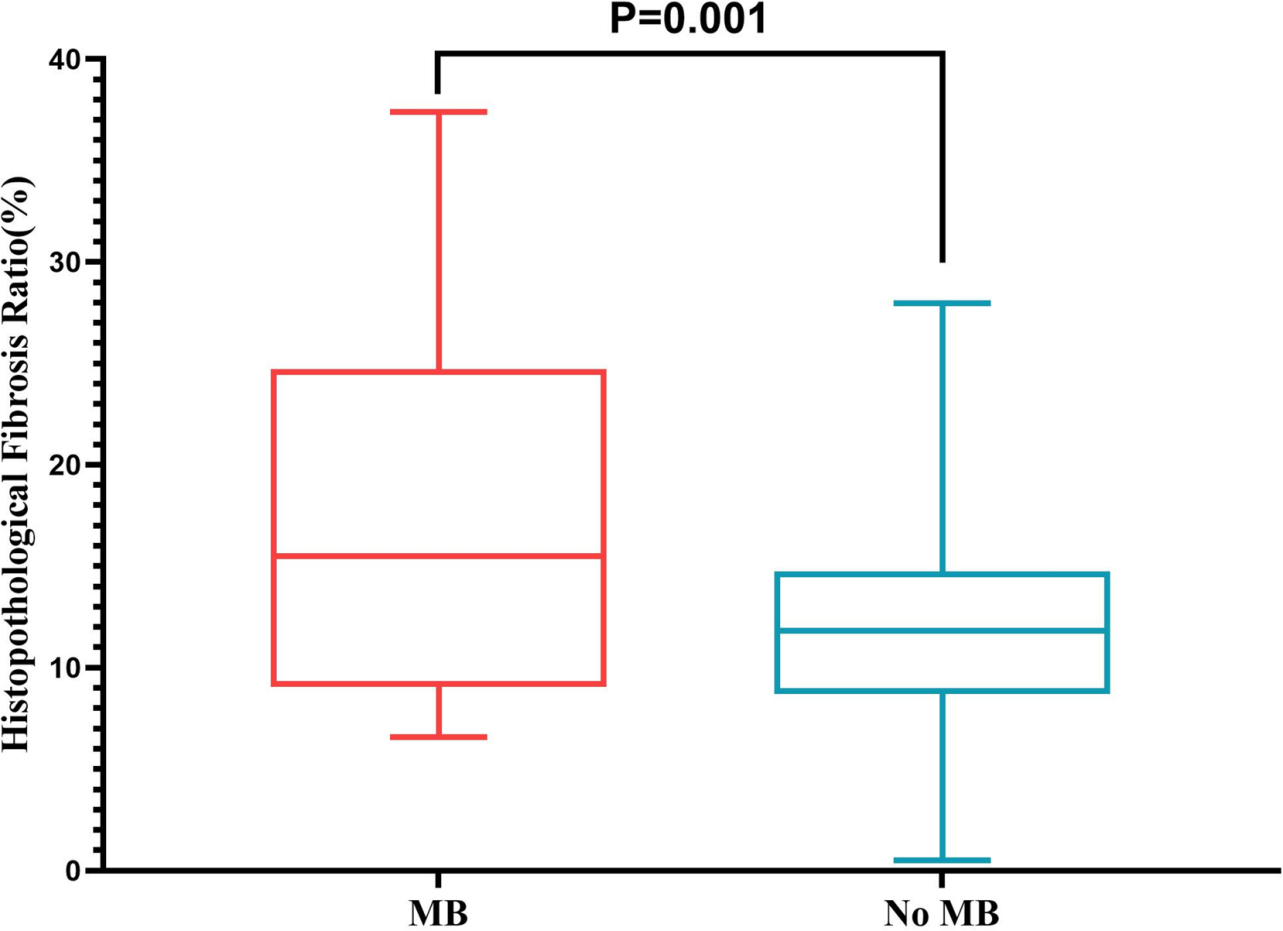
Histopathological myocardial fibrosis in the MB group. The histopathological myocardial fibrosis ratio was significantly higher in the MB group (17.0 ± 9.1% versus 12.2 ± 5.3%; *p* = 0.001)

Of these patients, 76 patients had MB. MB occurred mostly in the LAD (73/76), with an average systolic compression ratio of 77.5% and length of 26.7 mm (Table 2). Compared with patients without MB, those with MB were younger (37.2 ± 13.6 years vs. 47.2 ± 13.3 years, *p* < 0.001) and had less hypertension (7% vs. 22%, *p* = 0.002). Cardiac MRI analysis showed that patients with MB had a higher LGE mass index [8.7 (IQR: 4.0–22.4) g/m^2^ vs. 7.0 (IQR: 2.8–14.7) g/m^2^, *p* = 0.022] and global extent of LGE in the LV [10.3 (IQR: 5.1–19.1) %vs. 7.7 (IQR: 3.3–15.5) %, *p* = 0.032] than those without MB (Table 1). Furthermore, the histopathological analysis also showed a higher extent of myocardial fibrosis in patients with MB than in those without MB (17.0 ± 9.1% versus 12.2 ± 5.3%, *p* = 0.001) (Fig. 3).

**Table 2:** Characteristics of the myocardial bridging in this cohort

Variables	Values
No. of vessels involved, *n* (%)	
1 vessel	74 (97)
2 vessels	2 (3)
Location of MB, *n* (%)	
Proximal LAD	13 (17)
Middle LAD	59 (77)
Distal LAD	1 (1)
1st diagonal branch	1 (1)
LCX	1 (1)
PDA	3 (4)
Systolic compression ratio, %	77.5 ± 13.8
Length of MB, mm	26.7 ± 11.8
Depth of MB, mm	5.7 ± 1.5
Surgical treatment modalities, *n* (%)	
Myotomy	34 (45)
CABG	37 (49)

*CABG* Coronary artery bypass grafting, *LAD* Left anterior descending artery, *LCX* Left circumflex artery, *MB* Myocardial bridging, *PDA* Posterior descending artery

### Relationship of LGE and MB

Among the patients with MB ≥ 80%, 35 out of 43 were prescribed beta-blockers, and 4 out of 43 were prescribed calcium-channel blockers, while 37% (16 out of 43) of patients in this subgroup experienced stenocardial pain. There were no significant differences in N-terminal pro-brain natriuretic peptide (*n* = 69, 1761.8 ± 1240.0 vs. 1600.2 ± 875.9 pg/ml) and troponin I (*n* = 60, 0.07 ± 0.16 vs. 0.19 ± 0.66 pg/ml) between the two groups (MB ≥ 80% and < 80%).

**Fig. 4:**
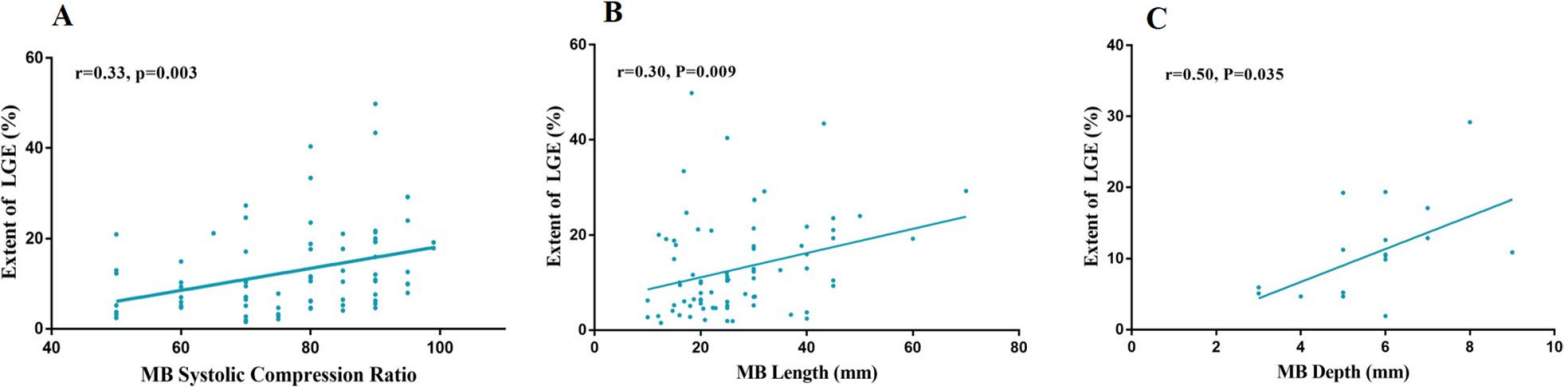
Correlation between the extent of LGE and the characteristics of MB. The global extent of LGE was correlated with the degree of systolic compression (*n* = 76) (**A**), length of tunneled segment (*n* = 76) (**B**), and depth of myocardial mass overlying the tunneled artery (*n* = 18) (**C**)

**Fig. 5:**
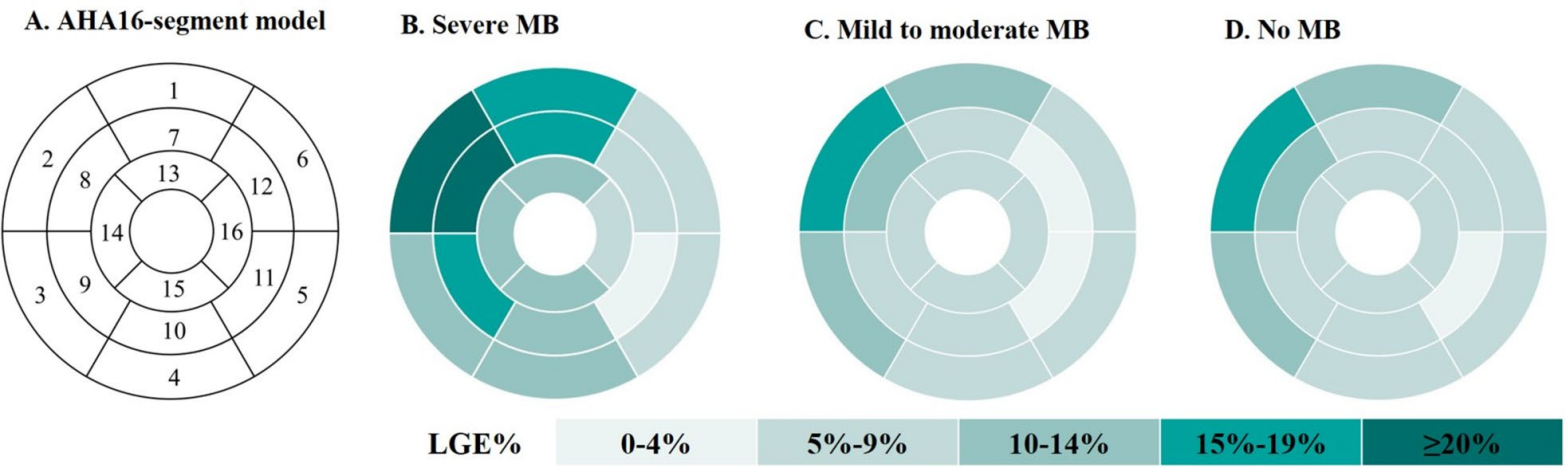
The distribution of LGE in this cohort. **A** The American Heart Association 16-segment model, 1 = basal anterior, 2 = basal anteroseptal, 3 = basal inferoseptal, 4 = basal lateral, 5 = basal inferolateral, 6 = basal anterolateral, 7 = mid anterior, 8 = mid anteroseptal, 9 = mid inferoseptal, 10 = mid inferior, 11 = mid inferolateral, 12 = mid anterolateral, 13 = apical anterior, 14 = apical septal, 15 = apical inferior, and 16 = apical lateral. **B** Distribution of the extent of LGE in patients with severe myocardial bridging. **C** Distribution of the extent of LGE in patients with mild to moderate myocardial bridging. **D** Distribution of the extent of LGE in patients without myocardial bridging

Further analysis of patients with MB showed that the global extent of LGE was correlated with the degree of systolic compression (*r* = 0.33, *p* = 0.003, *n* = 76), length of tunneled segment (*r* = 0.30, *p* = 0.009, *n* = 76), and depth of myocardial mass overlying the tunneled artery (*r* = 0.50, *p* = 0.035, *n* = 18) (Fig. 4). Multivariable linear regression analysis of LGE quantitation revealed that the degree of systolic compression was an independent risk factor for LGE (*β* = 0.292, *p* = 0.007) (Table 3). The extent of LGE was significantly higher in patients with severe MB than in those with mild to moderate MB [12.6% (IQR: 7.6–21.1%) vs. 5.9% (IQR: 3.0–11.2%), *p* = 0.0001]. The LGE fraction of basal anteroseptal and mid anteroseptal segments in patients with severe MB was significantly greater than that in patients with mild to moderate MB [basal anteroseptal: 23.1% (IQR: 14.9–33.5%) vs. 11.9% (IQR: 6.1–23.3%), *p* = 0.002; mid anteroseptal: 28.3% (IQR: 15.4–40.7%) vs. 5.2% (IQR: 2.3–15.5%), *p* = 0.001] and without MB [basal anteroseptal: 23.1% (IQR: 14.9–33.5%) vs. 14.7% (IQR: 6.1–28.3%), *p* = 0.003; mid anteroseptal: 28.3% (IQR: 15.4–40.7%) vs. 6.0% (IQR: 1.1–21.8%), *p* = 0.000] (Fig. 5).

**Table 3:** Linear regression analysis of the extent of LGE

Variables	Univariable analysis		Multivariable analysis		
	Beta	*p*-value	Beta	T	*p*-value
Age	-0.253	0.027	-0.015	-0.134	0.894
MB compression	0.333	0.003	0.292	2.759	0.007
MB length	0.297	0.009	0.143	1.338	0.185
LVEDVi	0.115	0.323	0.049	0.452	0.653
LV mass I	0.440	< 0.001	0.347	2.993	0.004

*LGE* Late gadolinium enhancement, *LVEDVi* LV End-diastolic volume index, *LV* *mass* *I*  left ventricular mass index, *MB* Myocardial bridging

### Prognostic value of MB in patients undergoing myectomy

**Fig. 6:**
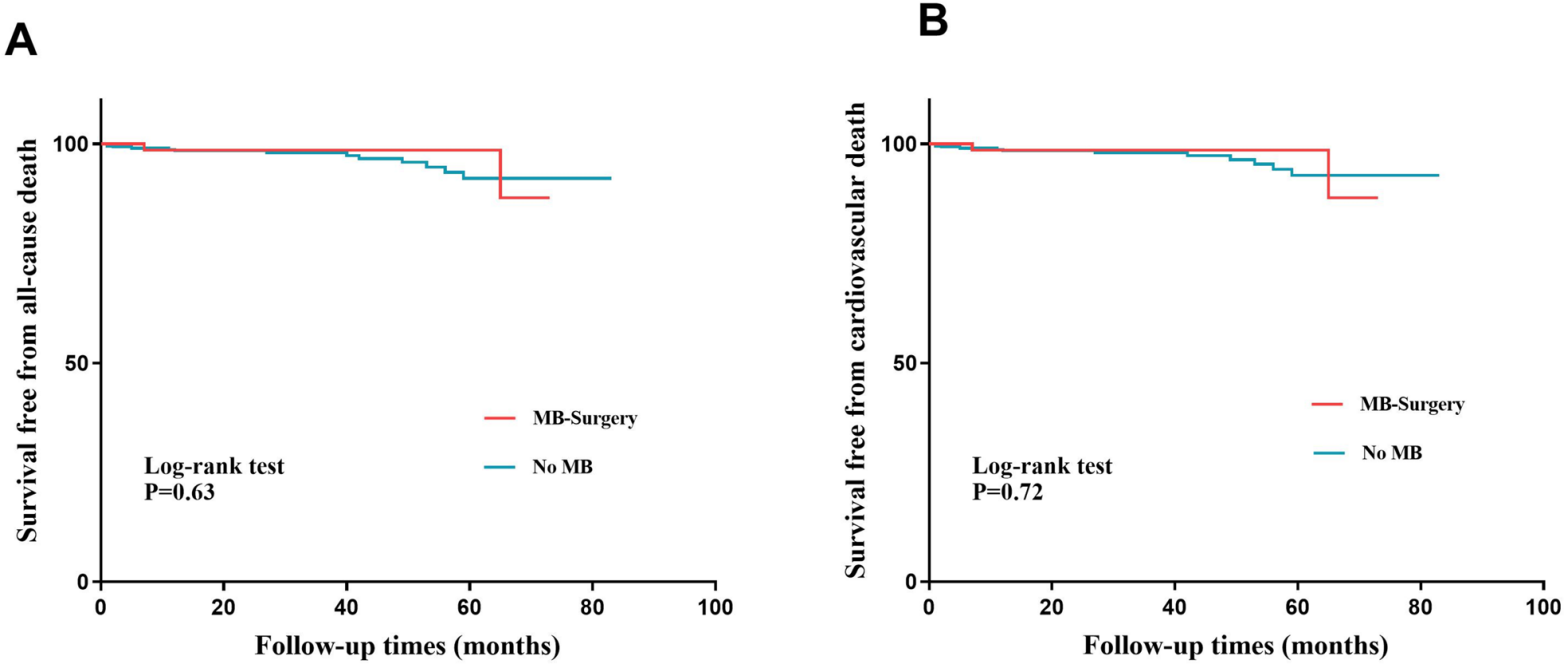
Kaplan–Meier analysis of the relationship of MB with survival free from all causes and cardiovascular mortality. Kaplan–Meier analysis did not identify a difference in all-cause death (**A**) or cardiovascular death (**B**) between patients with MB and those without MB

All the participants underwent surgical myectomy. Among the 76 patients with MB, 34 patients underwent myotomy, while 37 patients underwent coronary artery bypass grafting (CABG). During a median follow-up period of 28 (IQR: 15–52) months, 15 patients died (14 cardiac deaths). Kaplan–Meier analysis did not identify a difference in all-cause death (log-rank *p* = 0.63) or cardiovascular death (log-rank *p* = 0.72) between patients undergoing MB-related surgery and those without MB (Fig. 6).

## Discussion

The present study was designed to evaluate the role of myocardial bridging in myocardial fibrosis and prognosis in patients with overt obstructive HCM. The results demonstrated that the presence of myocardial bridging was associated with a higher extent of LGE. In addition, the degree of compression was independently linearly related to the extent of LGE in patients with myocardial bridging. However, myocardial bridging was not evidently associated with poor prognosis.

Increased myocardial fibrosis is a pathological hallmark of hypertrophic cardiomyopathy. Myocardial fibrosis in HCM is affected by multiple factors, including repetitive ischemia (i.e., blood supply–demand mismatch), hypertrophy, LVOT obstruction, and microvascular abnormalities [1–3]. Myocardial hypertrophy, microvascular dysfunction, and the remodeling and decreased density of intramural arterioles are very common in HCM. Previous studies showed correlations between the extent of LGE and maximal wall thickness and LVMI [13, 14]. Consistently, our data showed that the extent of LGE increased significantly with the indexed LV mass. However, the relationship between myocardial fibrosis and hypertrophy remains controversial because myocardial fibrosis could also be present in patients with genotype-positive/phenotype-negative HCM [15]. In addition, concomitant atherosclerotic coronary artery disease appears to exacerbate these abnormalities and worsen myocardial ischemia in patients with HCM [16].

Myocardial bridging is common in patients with HCM. In this study, the prevalence of myocardial bridging was 15.4%, which is similar to the data from other cohorts [5, 6]. MB was traditionally identified as a benign phenomenon. This was chiefly based on the observation that almost all coronary blood flow occurs during diastole, while MB is characterized by systolic arterial compression [4]. Furthermore, some studies demonstrated that the presence of myocardial bridging did not increase the risk of death in patients with HCM [7, 17]. However, a previous study demonstrated that transient MB compression could result in complex coronary flow patterns, including vortex rings, reverse flow, and recirculation regions, which can significantly alter the diastolic flow patterns and wall sheer stress distributions [9]. Putative mechanisms are complex. Intravascular ultrasound studies showed that systolic vessel compression could persist into the diastole period. The prolongation of the compression well into diastole is likely to compromise myocardial perfusion because the largest proportion of coronary blood flow normally occurs at this time [18]. The phenomenon could be drastically exaggerated in patients with obstructive HCM because of the presence of hyperdynamic systolic function and LVOTO with high intracavitary pressures.

A histological study found that hearts with myocardial bridging of the LAD had 33% (*p* = 0.0006) greater interstitial fibrosis than those without myocardial bridging [19]. However, the result may not be representative of the effect of myocardial bridging on fibrosis in HCM because the participants were without HCM. Cardiac MRI has been histologically proven to be a reliable method to measure myocardial replacement fibrosis and describe the distribution of myocardial fibrosis in HCM [13]. Our data showed that the extent of LGE was higher in patients with MB. Further analysis demonstrated an independent linear relationship between the compression ratio and the extent of LGE in patients with MB. As viewed from the American Heart Association 16-segment model, patients with severe myocardial bridging had a higher extent of LGE in basal to mid-anteroseptal segments and basal to mid-anterior segments than patients with mild-to-moderate or no myocardial bridging. These segments are supplied by LAD. Moreover, almost all the myocardial bridging in this study occurred in the proximal and middle segments of the LAD. This consistency further supports the correlation between severe myocardial bridging and the extent of LGE.

LGE provides valuable prognostic information in patients with HCM [1–3]. Some studies suggested that the presence of myocardial bridging was associated with a poor outcome and served as a risk factor for sudden death [18, 20]. One underlying mechanism may be that MB-related long-standing ischemia led to the formation of fibrosis, which was recognized as an arrhythmogenic substrate. Our study demonstrated for the first time the relationship between MB and fibrosis. Unfortunately, almost all the subjects with MB in this study underwent myotomy or coronary artery bypass grafting, which made it impossible to analyze the relationship between MB and clinical outcomes. However, MB-related surgery provided excellent postoperative survival similar to that of patients without MB, which could be partially explained by the surgical improvement of myocardial perfusion [6, 18]. Further serial studies are needed to illustrate (1) the relationship between MB and clinical outcomes and (2) the postoperative changes in LGE and myocardial ischemia in patients undergoing surgery for myocardial bridging.

Although the exact mechanism of the impact of myocardial bridging on fibrosis is unclear, our finding that severe MB is associated with increased fibrosis has implications for our better understanding of hypertrophic cardiomyopathy. Early detection of MB by coronary angiography and prescribing appropriate treatment modalities, including medicine and surgery, for patients with HCM could improve their clinical outcomes.

## Limitations

This study had limitations. First, this is a retrospective study, and only patients with preoperative CMR and coronary angiography were screened and enrolled, which could result in selection bias. Second, because examination of coronary flow reserve was not performed, we could not evaluate the correlation between LGE and coronary flow reserve. Third, the myocardial perfusion associated with MB was not well evaluated. This analysis could improve the management of MB in HOCM. Fourth, this study used digital subtraction angiography rather than computed tomography angiography to assess the MB, which could not fully characterize muscle bridges. Fifth, in terms of clinical outcomes, the small number of events during follow-up could affect the results to some extent. Sixth, almost all the subjects with MB in this study underwent myotomy or coronary artery bypass grafting, which made it impossible to analyze the relationship between MB and clinical outcomes. In the future, a well-designed prospective study is needed to obtain a better understanding of the impact of myocardial bridging on the clinical course of HCM.

## Conclusions

In conclusion, MB with severe systolic compression is significantly associated with a high extent of fibrosis in patients with obstructive HCM. This finding could expand our understanding of the influence of MB on the prognosis of HCM. In addition, concomitant myotomy or CABG might provide patients with MB with excellent survival similar to that of patients without MB.

## Data Availability

The datasets used and/or analyzed during the current study are available from the corresponding author on reasonable request.

## Abbreviations

HCM: Hypertrophic cardiomyopathy
LAD: Left anterior descending artery
LGE: Late gadolinium enhancement
LV: Left ventricular
LVOT: Left ventricular outflow tract
MB: Myocardial bridging

## References

[1] Ommen SR, Mital S, Burke MA, Day SM, Deswal A, Elliott P (2020). 2020 AHA/ACC Guideline for the Diagnosis and Treatment of Patients With Hypertrophic Cardiomyopathy: Executive Summary: A Report of the American College of Cardiology/American Heart Association Joint Committee on Clinical Practice Guidelines. Circulation.

[2] Maron BJ, Desai MY, Nishimura RA, Spirito P, Rakowski H, Towbin JA (2022). Diagnosis and evaluation of hypertrophic cardiomyopathy: JACC State-of-the-Art Review. J Am Coll Cardiol.

[3] Maron BJ (2018). Clinical course and management of hypertrophic cardiomyopathy. N Engl J Med.

[4] Sternheim D, Power DA, Samtani R, Kini A, Fuster V, Sharma S (2021). Myocardial bridging: diagnosis, functional assessment, and management: JACC State-of-the-Art Review. J Am Coll Cardiol.

[5] Nie C, Zhu C, Yang Q, Xiao M, Meng Y, Wang S (2021). Myocardial bridging of the left anterior descending coronary artery as a risk factor for atrial fibrillation in patients with hypertrophic obstructive cardiomyopathy: a matched case–control study. BMC Cardiovasc Disord.

[6] Bruce C, Ubhi N, McKeegan P, Sanders K (2023). Systematic review and meta-analysis of cardiovascular consequences of myocardial bridging in hypertrophic cardiomyopathy. Am J Cardiol.

[7] Tian T, Wang YL, Wang JZ, Sun K, Zou YB, Zhang WL (2014). Myocardial bridging as a common phenotype of hypertrophic cardiomyopathy has no effect on prognosis. Am J Med Sci.

[8] Elliott PM, Anastasakis A, Borger MA, Borggrefe M, Cecchi F, Charron P (2014). 2014 ESC guidelines on diagnosis and management of hypertrophic cardiomyopathy: the Task Force for the Diagnosis and Management of Hypertrophic Cardiomyopathy of the European Society of Cardiology (ESC). Eur Heart J.

[9] Sharzehee M, Chang Y, Song JP, Han HC (2019). Hemodynamic effects of myocardial bridging in patients with hypertrophic cardiomyopathy. Am J Physiol Heart Circ Physiol.

[10] Maron MS, Rowin EJ, Wessler BS, Mooney PJ, Fatima A, Patel P (2019). Enhanced American College of Cardiology/American Heart Association strategy for prevention of sudden cardiac death in high-risk patients with hypertrophic cardiomyopathy. JAMA Cardiol.

[11] Hashi AA, Ramesh Prasad GV, Connelly PW, Deva DP, Nash MM, Yuan W (2021). Cardiac MRI assessment of the right ventricle preand postkidney transplant. Int J Cardiovasc Imaging.

[12] Cerqueira MD, Weissman NJ, Dilsizian V, Jacobs AK, Kaul S, Laskey WK (2002). Standardized myocardial segmentation and nomenclature for tomographic imaging of the heart. A statement for healthcare professionals from the Cardiac Imaging Committee of the Council on Clinical Cardiology of the American Heart Association. Circulation..

[13] Liu J, Zhao S, Yu S, Wu G, Wang D, Liu L (2022). Patterns of replacement fibrosis in hypertrophic cardiomyopathy. Radiology..

[14] Zhang C, Liu R, Yuan J, Cui J, Hu F, Yang W (2016). Predictive values of N-terminal pro-B-type natriuretic peptide and cardiac troponin I for myocardial fibrosis in hypertrophic obstructive cardiomyopathy. PLoS One.

[15] Maron MS, Rowin EJ, Lin D, Appelbaum E, Chan RH, Gibson CM (2012). Prevalence and clinical profile of myocardial crypts in hypertrophic cardiomyopathy. Circ Cardiovasc Imaging.

[16] Sorajja P, Ommen SR, Nishimura RA, Gersh BJ, Berger PB, Tajik AJ (2003). Adverse prognosis of patients with hypertrophic cardiomyopathy who have epicardial coronary artery disease. Circulation.

[17] Basso C, Thiene G, Mackey-Bojack S, Frigo AC, Corrado D, Maron BJ (2009). Myocardial bridging, a frequent component of the hypertrophic cardiomyopathy phenotype, lacks systematic association with sudden cardiac death. Eur Heart J.

[18] Yetman AT, McCrindle BW, MacDonald C, Freedom RM, Gow R (1998). Myocardial bridging in children with hypertrophic cardiomyopathy–a risk factor for sudden death. N Engl J Med.

[19] Brodsky SV, Roh L, Ashar K, Braun A, Ramaswamy G (2008). Myocardial bridging of coronary arteries: a risk factor for myocardial fibrosis?. Int J Cardiol.

[20] Yetman AT, Hamilton RM, Benson LN, McCrindle BW (1998). Long-term outcome and prognostic determinants in children with hypertrophic cardiomyopathy. J Am Coll Cardiol.

